# Mesoporous Graphitic Carbon Nitrides Decorated with Cu Nanoparticles: Efficient Photocatalysts for Degradation of Tartrazine Yellow Dye

**DOI:** 10.3390/nano8090636

**Published:** 2018-08-21

**Authors:** Tao Zhang, Isis P. A. F. Souza, Jiahe Xu, Vitor C. Almeida, Tewodros Asefa

**Affiliations:** 1 Department of Chemical and Biochemical Engineering, Rutgers, The State University of New Jersey, 98 Brett Road, Piscataway, NJ 08854, USA; tz87@scarletmail.rutgers.edu (T.Z.); jx141@scarletmail.rutgers.edu (J.X.); 2 Laboratory of Environmental and Agrochemistry, Department of Chemistry, State University of Maringá, 5790 Colombo Avenue, Maringá, Paraná 87020-900, Brazil; isispelegrini@hotmail.com; 3 Department of Chemistry and Chemical Biology, Rutgers, The State University of New Jersey, 610 Taylor Road, Piscataway, NJ 08854, USA

**Keywords:** mesoporous graphitic carbon nitride, copper nanoparticles, photodegradation, tartrazine yellow, photocatalysis

## Abstract

A series of mesoporous graphitic carbon nitride (mpg-C_3_N_4_) materials are synthesized by directly pyrolyzing melamine containing many embedded silica nanoparticles templates, and then etching the silica templates from the carbonized products. The mass ratio of melamine-to-silica templates and the size of the silica nanoparticles are found to dictate whether or not mpg-C_3_N_4_ with large surface area and high porosity form. The surfaces of the mpg-C_3_N_4_ materials are then decorated with copper (Cu) nanoparticles, resulting in Cu-decorated mpg-C_3_N_4_ composite materials that show excellent photocatalytic activity for degradation of tartrazine yellow dye. The materials’ excellent photocatalytic performance is attributed to their high surface area and the synergistic effects created in them by mpg-C_3_N_4_ and Cu nanoparticles, including the Cu nanoparticles’ greater ability to separate photogenerated charge carriers from mpg-C_3_N_4_.

## 1. Introduction

The fast-growing global economy has resulted in a vast number of industries, many of which directly or indirectly generate various environmental pollutants, which can cause serious threats to society’s well-being and the development of a sustainable future [1]. Therefore, there is no question that these pollutants must be tackled to overcome their many undesirable consequences. Among many remediation strategies to address environmental pollutants, semiconductor-based solar photocatalysis, which utilizes the abundant solar energy irradiated by the sun to decompose environmentally polluting organic and inorganic species via various light-induced redox reactions over semiconductor materials, is quite promising [2]. While many semiconducting materials, such as TiO_2_, ZnO, and SnO_2_, have been extensively studied for this purpose, their large band gap energy hinders the absorption of visible light and results in inefficient utilization of solar energy to photocatalyze reactions over these materials.

Graphitic carbon nitride (g-C_3_N_4_), a low-cost metal-free polymeric semiconductor with great thermal and chemical stability and good electronic structure and photoactivity in the visible region of electromagnetic radiation, has recently emerged as a promising visible-light photocatalyst for degradation of various pollutant using sunlight, and thus stimulated intensive research works [2,3]. g-C_3_N_4_ materials are often synthesized by treating certain nitrogen-rich organic precursors at high-temperature. However, g-C_3_N_4_ materials synthesized via high-temperature thermal treatment of precursors such as cyanamide, dicyandiamide, melamine, thiourea and urea generally possess non-porous structure and relatively low surface area because these precursors tend to undergo a high degree of polycondensation at high temperature [4,5]. Thus, finding synthetic methods and/or precursors that can lead to advanced g-C_3_N_4_ materials with high surface area and large porosity is of paramount importance, since these structural features provide the materials with more accessible catalytically active sites and better pathways for mass transport of reactants and products and thus improved photocatalytic performances for decontamination of pollutants, water splitting, carbon dioxide reduction, etc. [2,6,7]. 

Meanwhile, although g-C_3_N_4_ has a good band gap energy (2.7 eV) for visible-light photocatalysis, with conduction band (CB) and valence band (VB) potentials located at ca. –1.1 eV and ca. +1.6 eV vs normal hydrogen electrode (NHE), respectively [5], its photo-induced electron-hole pairs still suffer from rapid recombination even when high surface area and large porosity are introduced into the material. As a result, g-C_3_N_4_‘s charge carriers are often unable to participate in many photocatalytic reactions and the material shows poor photocatalytic activity [1]. In order to improve charge separation as well as light absorption over g-C_3_N_4_, many strategies have been developed. For example, some improvement in the optical properties and electronic structures of g-C_3_N_4_ have been realized by depositing metal particles on it [8], doping or co-doping heteroatoms (metals or nonmetals) on it [6,9,10], combining it with carbon materials [11] and constructing heterojunctions over its structures [12]. In the case of metal deposition, the immobilized metal particles on the surfaces of g-C_3_N_4_ increase charge separation, usually by capturing the photogenerated electrons and preventing them from combining with the holes. For this, noble metals such as Au [13], Pt [14], Pd [15] and Ag [16] have particularly been used, and their hybrid products with g-C_3_N_4_ have shown superior photocatalytic performances. Compared with noble metals, non-noble metals such as Cu are more appealing to use for these purposes because they are more Earth-abundant and can be combined with g-C_3_N_4_ to yield low-cost hybrid materials for a broad range of applications [17,18]. In terms of the potential applications of such g-C_3_N_4_-based semiconductor composite materials, pollutant degradation is of particular interest. In fact, g-C_3_N_4_-based materials have been demonstrated to degrade a variety of organic contaminants, including methyl orange, methylene blue and rhodamine B [5].

Over the past few years, melamine has been successfully used as precursor to synthesize g-C_3_N_4_ materials by thermal condensation [19,20]. Melamine has some advantages, especially compared with cyanamide precursors that are more commonly used to make g-C_3_N_4_. It is nontoxic and inexpensive. Furthermore, it is less likely to produce toxic and flammable byproducts if additional solution-phase nanocasting synthetic steps, e.g., rigid template materials (such as mesoporous or colloidal SiO_2_) to create nanopores in g-C_3_N_4_, are included [4].

Herein, we show that melamine and colloidal silica nanoparticles with different sizes can be used, as precursor and hard templates, respectively, to produce mesoporous g-C_3_N_4_ (mpg-C_3_N_4_) materials with three-dimensionally interconnected frameworks, large surface areas and high porosities. The mass ratio of melamine-to-silica nanoparticles is found to be crucial in the formation of high surface area and desirable pore sizes in the final g-C_3_N_4_ materials. The pore size in the materials was easily tailored (to be around 12, 22, 47 or 79 nm) simply by changing the sizes of the silica nanoparticles used as templates during the synthesis. By functionalizing the surfaces of the mpg-C_3_N_4_ materials with Cu nanoparticles, mpg-C_3_N_4_ materials decorated with Cu nanoparticles are produced. The Cu-decorated mpg-C_3_N_4_ composite material are then demonstrated, for the first time, to serve as efficient photocatalysts for degradation of tartrazine yellow, a colorful azo-dye that is widely used as a colorant in various food and pharmaceutical products in many countries, but is also implicated in causing allergies, hyperactivity and even cancer if consumed in excess, and is banned in some countries as a result [21]. 

## 2. Experimental Section

### 2.1. Materials, Chemicals and Reagents

Colloidal silica dispersions (12 nm, Ludox TM-40, 40 wt.% suspension in water and 22 nm, Ludox HS-40, 40 wt.% suspension in water), copper(II) nitrate hemi(pentahydrate) and sodium borohydride were obtained from Sigma-Aldrich (St. Louis, MO, USA). Colloidal silica dispersions (47 nm, SNOWTEX-30LH, 30 wt.% suspension in water and 79 nm, SNOWTEX-ZL, 40 wt.% suspension in water) were purchased from Nissan Chemical America Corporation (Houston, TX, USA). Melamine (99%) and ammonium hydrogen difluoride (95%) were acquired from Alfa Aesar (Haverhill, MA, USA). Tartrazine yellow was obtained from Duas Rodas Company (Campinas, São Paulo, Brazil). All chemicals and reagents were of analytical grade and used as received without further purification. Distilled water was used throughout the experiments.

### 2.2. Synthesis of Melamine-Derived Mesoporous g-C_3_N_4_ (mpg-C_3_N_4_)

First, melamine (1 g, 7.92 mmol) and different amounts of 12 nm colloidal silica (0.25, 1, 2 or 4 g) were mixed with hot water (80 °C, 80 mL), and the solution was stirred for 1 h at 80 °C in a closed beaker. The beaker was then left open, and the solution was kept at 100 °C to let the solvent in it evaporate completely. The resulting composite materials comprising melamine-silica nanoparticles were pyrolyzed in a semi-closed or a partially covered combustion boat in a tube furnace under argon atmosphere at 550 °C for 3 h at a ramp of 20 °C min^−1^. In order to create nanoporous structures in the materials, the silica templates were removed from the resulting yellow powders by treating them in 4 M ammonium hydrogen difluoride solution for 24 h at room temperature. The solid products were recovered via filtration, washed with copious amount of water and then ethanol, and finally kept at 60 °C to dry. The resulting materials were denoted as 12-mpg-C_3_N_4_−*x*, where *x* represents the initial mass ratio of melamine-to-colloidal silica templates, which is 0.25, 0.5, 1 or 4. As a control material for the studies, bulk g-C_3_N_4_ was prepared without including 12 nm silica nanoparticles templates in the precursor. In addition, by using colloidal silica templates with sizes of 22, 47 or 79 nm, but keeping the melamine-to-silica nanoparticles ratio as 1:1 and the synthetic procedures and conditions the same, three other mesoporous g-C_3_N_4_ materials, named *y*-mpg-C_3_N_4_-1, where *y* represents the size of colloidal silica templates (22, 47 or 79 nm), were synthesized. 

### 2.3. Synthesis of Cu Nanoparticles-Decorated 22-mpg-C_3_N_4_-1 Materials

Two Cu-decorated 22-mpg-C_3_N_4_-1 materials containing different amounts of Cu nanoparticles were synthesized by mixing two different concentrations of aqueous Cu^2+^ solutions (5 or 10 wt.% Cu^2+^) with 22-mpg-C_3_N_4_-1, followed by the reduction of the Cu^2+^ ions with NaBH_4_ solution. First, 22-mpg-C_3_N_4_-1 (300 mg) was dispersed in water (40 mL) containing Cu^2+^ ions (15 mg, 0.23 mmol), and then a desired amount of NaBH_4_ (50 mg, 1.32 mmol) was slowly added into the mixture. The mixture was stirred for 4 h at room temperature and then filtered. The solid product was washed with water and then ethanol and dried in an oven at 60 °C overnight. The product had 5 wt.% Cu and was named as 5Cu-22-mpg-C_3_N_4_-1. The corresponding material containing 10 wt.% Cu was obtained in a similar manner but using a higher amount of Cu^2+^ ions (30 mg, 0.47 mmol). The material was named 10Cu-22-mpg-C_3_N_4_-1. 

### 2.4. Characterization

The Brunauer–Emmett–Teller (BET) surface areas and pore properties of the materials were analyzed by N_2_ porosimetry (at −196 °C) using a Tristar-3000 instrument (Micromeritics Instrument Corporation, Norcross, GA, USA). Before each measurement, the sample was degassed at 80 °C for 12 h under a flow of N_2_ gas to remove any possible gas impurities adsorbed on the sample’s surfaces. Based on the adsorption–desorption data and isotherm, the surface areas and the pore size distributions were calculated with the Brunauer–Emmett–Teller (BET) method and the Barrett–Joyner–Halenda (BJH) method, respectively. Transmission electron microscope (TEM) images of the materials were taken with a JEOL 1200EX transmission electron microscope (JEOL Inc., Akishima, Tokyo, Japan). The powder X-ray diffraction (XRD) patterns of the materials were obtained with a Philips X’Pert XRD diffractometer (Philips, Almelo, the Netherlands) operating with Cu Kα radiation as the X-ray source. The XRD patterns were recorded in a 2*θ* range between 10° and 80° with a step size (2*θ*) of 0.02° and a scan rate of 0.6° min^−1^. Thermogravimetric analyses of the materials were conducted with a thermogravimetric analysis (TGA7) instrument (PerkinElmer, Waltham, MA, USA) at a heating rate of 5 °C min^−1^ and under a flow of air passing over the samples at a rate of 20 mL min^−1^. The UV-Vis diffuse reflectance spectra (UV-Vis DRS) of the materials in a spectral range of 250–800 nm were obtained with a Lambda 950 spectrophotometer (PerkinElmer, Waltham, MA, USA). Small angle X-ray scattering (SAXS) patterns of the materials were acquired using a Brüker AXS HI-STAR Area Detector Diffraction system (Brüker, Billerica, MA, USA) equipped with an Enraf-Nonius FR-571 rotating anode X-ray generator operating with a graphite monochromator (Cu K_α_; λ = 1.5418 Å). The surface chemical compositions of the materials were analyzed with a Thermo Scientific K-Alpha X-ray photoemission spectrometer (XPS) (Thermo Fisher Scientific, Waltham, MA, USA) operating with Al K_α_ X-ray source (hv = 1486.6 eV). For high-resolution spectra of individual peaks, the scans were performed with an energy resolution of 0.1 eV. Energy dispersive X-ray fluorescence (EDXRF) was performed using DX-95 EDXRF spectrometer (Thermo Fisher Scientific, Waltham, MA, USA) equipped with Rh tube.

### 2.5. Photocatalytic Degradation of Tartrazine Yellow Dye

The photocatalytic performances of the as-prepared mpg-C_3_N_4_ materials containing different loadings of Cu nanoparticles as well as the control material bulk C_3_N_4_ for degradation of tartrazine dye in solutions were evaluated by subjecting the samples to UV light emitted by a Germicide lamp, 18 W (Osram, Münich, Germany) in a jacketed beaker at room temperature, as in our previous report [21]. Blank experiments were also performed without putting the photocatalysts in the solution. Typically, prior to the test, 100 mg of the catalyst (with a concentration of 0.5 g L^−1^) was mixed with 200 mL tartrazine yellow dye solution (with a concentration of 10 mg L^−1^). The mixture was stirred for 30 min in the dark to let the solution and the catalyst reach adsorption–desorption equilibrium. The light was then turned on. During photocatalytic reaction, 4.0 mL of a suspension was sampled every 30 min using 0.45 µm syringe filter (MilliporeSigma, Burlington, MA, USA), and the concentration of tartrazine yellow in the supernatant was measured and quantified using Cary 50 UV-Vis spectrophotometer (Agilent Technologies, Santa Clara, CA, USA). 

## 3. Results and Discussion

**Scheme 1:**
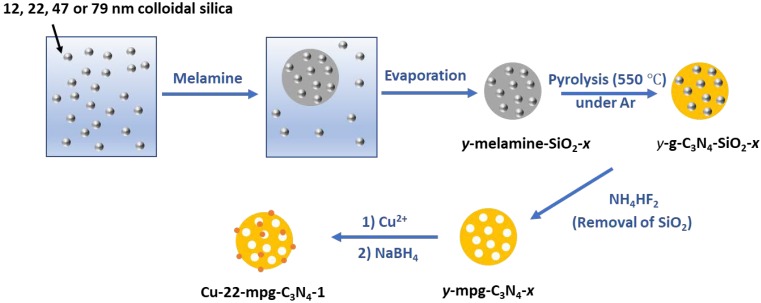
Schematic illustration of the procedure used to synthesize *y*-mpg-C_3_N_4_-*x* and Cu-22-mpg-C_3_N_4_-1 materials, where *y* and *x* represent the size of the silica nanoparticles used as templates and the mass ratio of melamine-to-silica nanoparticles used for the synthesis of the materials, respectively.

The synthetic procedures that were employed to make the set of mesoporous C_3_N_4_ and Cu-decorated mesoporous C_3_N_4_ materials (denoted as *y*-mpg-C_3_N_4_-*x* and Cu-22-mpg-C_3_N_4_ respectively) are illustrated in Scheme 1 and also described in the Experimental section above. First, an optimal mass ratio of melamine-to-silica nanoparticles that can render the material with high surface area and desired pore sizes, replicating the sizes of the silica nanoparticles templates, was determined. Briefly, melamine was mixed with different amounts of 12 nm colloidal silica templates and the solvent was evaporated resulting in melamine/silica nanoparticles composite materials. After subjecting the composite materials to thermal condensation and removing the silica templates from the carbonized composite products, a set of 12-mpg-C_3_N_4_−*x* materials was obtained, in which *x* represents the mass ratio of melamine-to-silica nanoparticles used (0.25, 0.5, 1, or 4). Another set of materials, *y*-mpg-C_3_N_4_-1, where *y* represents the size of colloidal silica templates, was synthesized using 22, 47 or 79 nm silica nanoparticles as templates with an optimized *x* value (*x* = 1). Two different amounts of Cu^2+^ (5 or 10 wt.%) were then deposited onto the surfaces of one of the mpg-C_3_N_4_ materials, 22-mpg-C_3_N_4_-1. After reducing the Cu^2+^ ions with NaBH_4_, photocatalytically active Cu-modified mpg-C_3_N_4_ materials, denoted 5Cu-22-mpg-C_3_N_4_-1 and 10Cu-22-mpg-C_3_N_4_-1, respectively, were obtained.

**Figure 1:**
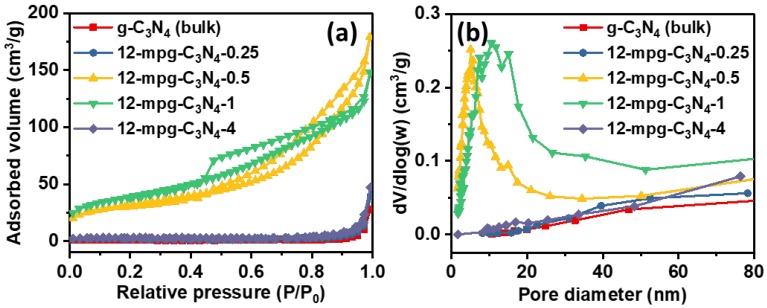
(**a**) N_2_ adsorption–desorption isotherms of 12-mpg-C_3_N_4−*x*_ materials synthesized using different mass ratios of melamine-to-silica nanoparticles and (**b**) their corresponding pore size distributions.

The structures of 12-mpg-C_3_N_4_−*x* were first investigated by N_2_ porosimetry. The results showed that the surface area and porosity of the materials depended on the initial mass ratio of melamine-to-colloidal silica (12 nm) templates used to synthesize the materials (Figure 1). Bulk g-C_3_N_4_, which was synthesized without using colloidal silica templates, exhibited low surface area (3 m^2^/g) and non-porous structure (0.04 cm^3^/g). When colloidal silica nanoparticles templates were used, the surface area of the materials first increased, from 7 m^2^/g for 12-mpg-C_3_N_4_-0.25 (which was synthesized using melamine-to-silica nanoparticles mass ratio of 0.25) to 136 m^2^/g for 12-mpg-C_3_N_4_-1 (which was synthesized using melamine-to-silica nanoparticles mass ratio of 1), but then decreased to 9 m^2^/g for 12-mpg-C_3_N_4_-4 (which was synthesized using the highest ratio of melamine-to-silica nanoparticles of 4). The pore volume of the materials exhibited a similar trend, namely, it first increased from 0.06 cm^3^/g for 12-mpg-C_3_N_4_-0.25 to 0.27 cm^3^/g for 12-mpg-C_3_N_4_-1, but then decreased to 0.05 cm^3^/g for 12-mpg-C_3_N_4_-4). It is also worth noting that only 12-mpg-C_3_N_4_-1 (the pore sizes of which are centered at ca. 11.7 nm) showed pores close to the size of colloidal silica templates used to synthesize the material. Low or high melamine-to-silica mass ratio (i.e., *x* = 0.25 or 4) did not result in materials the pore size of which directly correspond to each silica nanoparticle; in both cases, the materials were largely non-porous and had a small amount of large pores. 

**Scheme 2:**
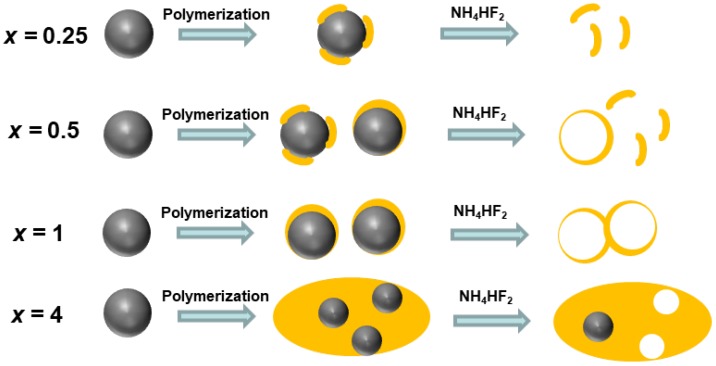
Proposed processes leading to different 12-mpg-C_3_N_4_-*x* materials from melamine/silica nanoparticles composite precursors containing different mass ratios of melamine-to-silica nanoparticles.

Based on N_2_ porosimetry results presented above, the processes leading to the formation of mpg-C_3_N_4_ and Cu-loaded mpg-C_3_N_4_ were proposed as illustrated in Scheme 2. For the lowest mass ratio of melamine-to-silica nanoparticles (*x* = 0.25, where the mass of silica nanoparticles is much higher than that of melamine), the carbonized material does not maintain its porous structure after removal of silica due to the insufficient amount of melamine and the small amount of carbon product forming from it, which can easily collapse. The small proportion of large pores in it seem to replicate some aggregated silica nanoparticles templates. For the highest mass ratio of melamine-to-silica nanoparticles (*x* = 4), the lack of many silica nanoparticles templates can inhibit the formation of carbon structures around the particles and thereby the formation of porous carbon structure in the material. In addition, the formation of compact g-C_3_N_4_ shells around the silica spheres templates, due to the relatively higher amount of melamine used for the synthesis, can prevent ammonium hydrogen difluoride from reaching the silica templates and etching them all. As a result, the silica templates cannot all be removed from the carbonized product and porous structures cannot be formed in this material. This was actually corroborated by thermogravimetric analysis (TGA) (Figure S1), which clearly revealed that 12-mpg-C_3_N_4_-4 had higher amount of residue associated with silica (ca. 6.79 wt.%) compared with 12-mpg-C_3_N_4_-1 (ca. 1.12 wt.%). However, the low surface area in the former was likely to be caused also by the lack of porous structure in the material, since most of the silica was removed, despite a large amount of it being left behind, and yet the material showed low surface area. Based on the above results, the optimal mass ratio of melamine-to-silica nanoparticles for the synthesis of high surface area mpg-C_3_N_4_ materials with desired pore sizes, i.e., pores corresponding to the size of the silica nanoparticles, was determined to be around 1. 

**Figure 2:**
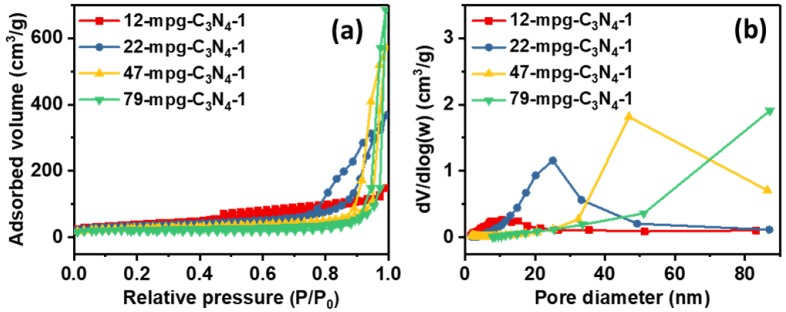
(**a**) N_2_ adsorption–desorption isotherms of different *y*-mpg-C_3_N_4_-1 materials synthesized from melamine/silica nanoparticles composite precursors containing different mass ratios of melamine-to-silica nanoparticles and (**b**) their corresponding pore size distributions.

The synthetic procedure was easily extended to produce a series of *y*-mpg-C_3_N_4_-1 materials with different pore sizes (with *y* = 12, 22, 47 or 79 nm, representing the different sizes of silica nanoparticles used as templates to create the pores) (Figure 2). When the sizes of the silica nanoparticles templates used to synthesize the materials were increased, the surface areas of the *y*-mpg-C_3_N_4_-1 materials slightly decreased (from 136 m^2^/g to 73 m^2^/g), but their corresponding pore volumes increased (from 0.27 cm^3^/g to 1.05 cm^3^/g). Quantitative results obtained by N_2_ porosimetry analyses of the materials are compiled in Table 1 and Table 2.

**Figure 3:**
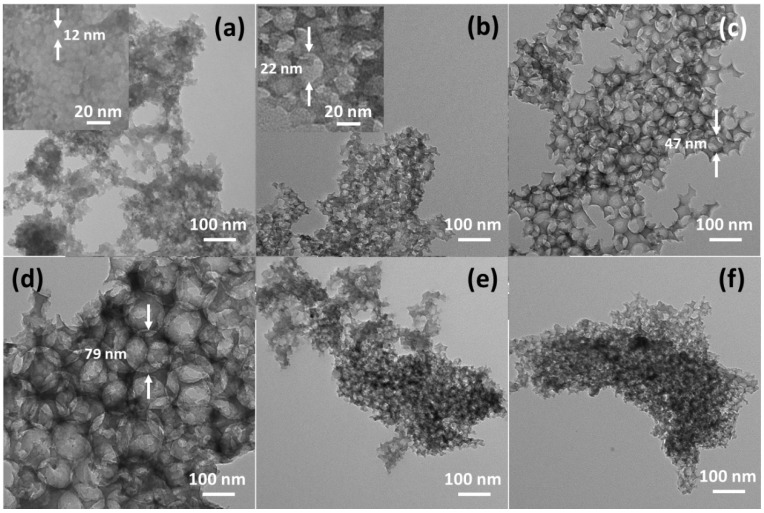
Transmission electron microscope (TEM) images of the series of *y*-mpg-C_3_N_4_-1 and Cu-22-mpg-C_3_N_4_-1 materials synthesized using different sizes of silica nanoparticles as templates and modified with different loadings of Cu: (**a**) 12-mpg-C_3_N_4_-1; (**b**) 22-mpg-C_3_N_4_-1; (**c**) 47-mpg-C_3_N_4_-1; (**d**) 79-mpg-C_3_N_4_-1; (**e**) 5Cu-22-mpg-C_3_N_4_-1; and (**f**) 10Cu-22-mpg-C_3_N_4_-1.

The structures of *y*-mpg-C_3_N_4_-1 materials were further examined by transmission electron microscope (TEM) (Figure 3a–d). When *x* = 1, the material showed pores with sizes of ca. 12 nm (Figure 3a), corroborating the pore size obtained by N_2_ porosimetry for this material (12-mpg-C_3_N_4_-1) earlier (Table 1). The TEM images of the *y*-mpg-g-C_3_N_4_-1 materials, which were synthesized by keeping the same *x* value but by changing the sizes of the silica nanoparticles templates, clearly showed that all the materials possessed nanoporous structures. However, their pore sizes were highly dependent on the sizes of the silica templates used to synthesize them, and generally increased as the sizes of colloidal silica templates were increased from 12 nm to 79 nm. In addition, TEM images were also acquired for 5Cu-22-mpg-C_3_N_4_-1 and 10Cu-22-mpg-C_3_N_4_-1 (Figure 3e,f). The images of these materials showed many dark spots or areas associated with Cu species. Overall, the N_2_ porosimetry and TEM results above clearly indicated that mesoporous g-C_3_N_4_ materials with high surface area and mesopore sizes could be produced by a facile synthetic method using melamine as precursor and a proper amount of silica nanoparticles as templates. Furthermore, the pore sizes in the materials could be tuned by varying the sizes of the silica nanoparticles used as templates to synthesize the materials.

**Figure 4:**
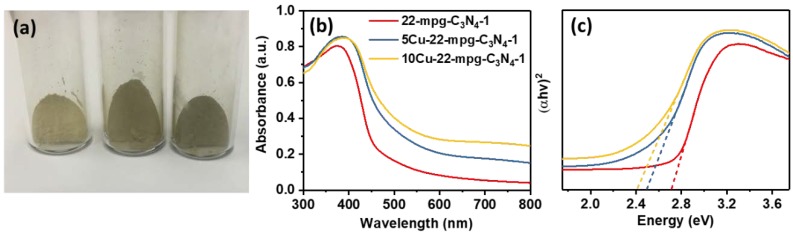
(**a**) Physical appearance of 22-mpg-C_3_N_4_-1, 5Cu-22-mpg-C_3_N_4_-1, and 10Cu-22-mpg-C_3_N_4_-1 materials (from left to right); (**b**) UV-Vis diffuse reflectance spectra (DRS) of 22-mpg-C_3_N_4_-1, 5Cu-22-mpg-C_3_N_4_-1, and 10Cu-22-mpg-C_3_N_4_-1 and (**c**) the corresponding plots of transformed Kubelka–Munk function versus the energy of incident light.

Since 22-mpg-C_3_N_4_-1 and Cu-22-mpg-C_3_N_4_-1 were used as representative materials to evaluate the photocatalytic activity of Cu-doped mpg-C_3_N_4_ materials for degradation of tartrazine yellow dye, the following characterizations were carried out only for these two materials. Upon increasing the relative amount of Cu^2+^ ions used to synthesize the different Cu-22-mpg-C_3_N_4_-1 materials, the colors of the samples slowly changed from yellow to light green (Figure 4a). UV-Vis diffuse reflectance spectroscopy (DRS) was used to determine the optical properties of these materials between 250 nm to 800 nm (Figure 4b). The intrinsic absorption edge of 22-mpg-C_3_N_4_-1 was found to be ca. 459 nm, which is in line with the value reported for g-C_3_N_4_ materials in the literature [22]. After decorating its surfaces with 5 wt.% and 10 wt.% of Cu (producing 5Cu-22-mpg-C_3_N_4_-1 and 10Cu-22-mpg-C_3_N_4_-1 materials), the absorption edge shifted to higher wavelengths, to ca. 498 nm and ca. 514 nm, respectively, and the intensity of the absorption band increased; this suggests that the modification of mpg-C_3_N_4_ with Cu, at least, up to 10 wt.% enhances the material’s ability to absorb visible light [23].

The band gap energy (*E*_g_) of the materials were calculated based on the spectra obtained with UV-Vis DRS and using the transformed Kubelka–Munk equation (Figure 4b): ${\left[F\left(R\right)\cdot \mathrm{hv}\right]}^{2}{=\alpha =A(\mathrm{hv}-E}_{g})$, where $F\left(R\right)={\left(1-R\right)}^{2}/2R$, where hv is the incident photon energy, R is the percentage of reflected light, $E_{g}$ is the band gap energy and A is constant, the value of which depends on the transition probability [24,25]. The value of $E_{g}$ for 22-mpg-C_3_N_4_-1 was determined to be 2.71 eV, whereas the values of $E_{g}$ for 5Cu-22-mpg-C_3_N_4_-1 and 10Cu-22-mpg-C_3_N_4_-1 were determined to be 2.49 and 2.41 eV, respectively. This result indicates that the value of $E_{g}$ decreases as the amount of Cu in mpg-C_3_N_4_-1 is increased. These results are consistent with those reported for g-C_3_N_4_ in the literature, where a steady decrease in band gap energy is observed as more metal is loaded on g-C_3_N_4_ [26,27,28]. Overall, the DRS results suggested that Cu/mpg-C_3_N_4_ composite materials have significantly improved light absorption; they can thus be potentially better photocatalysts compared with pure g-C_3_N_4_.

**Figure 5:**
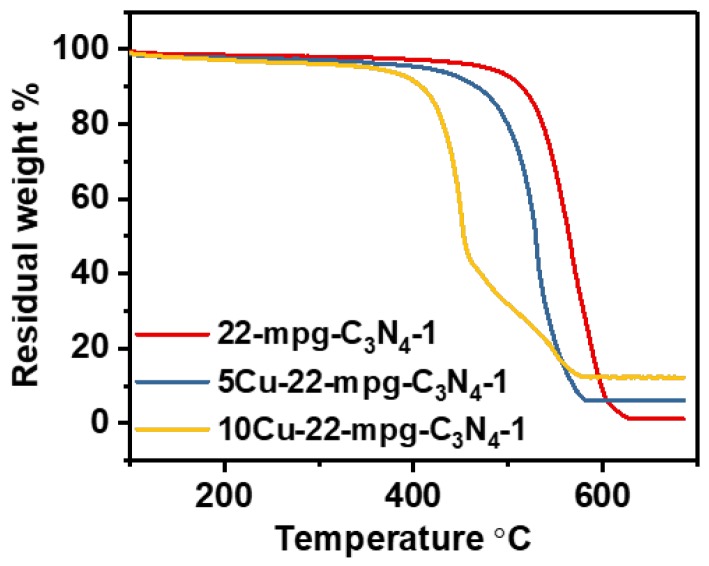
Thermogravimetric analysis (TGA) curves obtained in air for 22-mpg-C_3_N_4_-1, 5Cu-22-mpg-C_3_N_4_-1 and 10Cu-22-mpg-C_3_N_4_-1 materials.

The amount of Cu in Cu-22-mpg-C_3_N_4_-1 was estimated by comparing the weight of the residue that remained in Cu-mpg-C_3_N_4_ at the end of thermogravimetric analysis (TGA) in air with respect to that of 22-mpg-C_3_N_4_-1 (Figure 5). While 22-mpg-C_3_N_4_-1 gave ca. 1.12 wt.% residue, which is associated with residual silica, since the C_3_N_4_ in it should be lost in the form of CO_2_ and NO_2_ during calcination, 5Cu-22-mpg-C_3_N_4_-1 and 10Cu-22-mpg-C_3_N_4_-1 gave ca. 6.07 wt.% and 12.24 wt.% residue, which should be largely due to CuO. From these values, the amounts of Cu in 5Cu-22-mpg-C_3_N_4_-1 and 10Cu-22-mpg-C_3_N_4_-1 were determined to be ca. 3.73 wt.% and 8.68 wt.%, respectively. In addition, it is worth noting that the presence of Cu NPs in mpg-C_3_N_4_ facilitated the materials’ thermal decomposition. While 22-mpg-C_3_N_4_-1 was stable up to ca. 480 °C in air and decomposed only in the range of 480–625 °C, the Cu-functionalized counterparts, 5Cu-22-mpg-C_3_N_4_-1 and 10Cu-22-mpg-C_3_N_4_-1, decomposed in lower temperature ranges of 440–575 °C and 390–560 °C, respectively. Furthermore, the decreased thermal stability of Cu-22-mpg-C_3_N_4_-1 materials compared with mpg-C_3_N_4_-1 indirectly indicated the successful decoration of the surfaces of mpg-C_3_N_4_ with Cu nanoparticles. This is not unprecedented as the presence of Cu (metallic) particles has been reported to facilitate heat transfer during calcination and promote the combustion of g-C_3_N_4_ [29].

**Figure 6:**
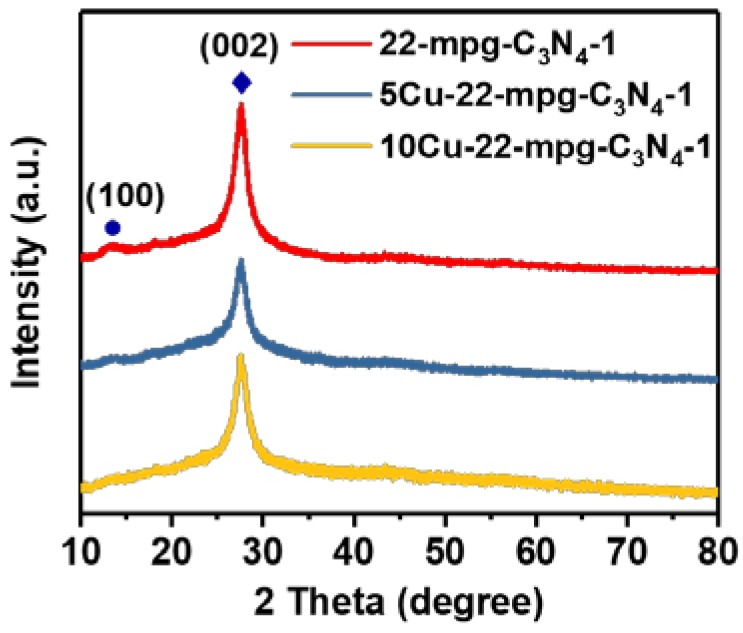
X-ray diffraction (XRD) patterns of 22-mpg-C_3_N_4_-1, 5Cu-22-mpg-C_3_N_4_-1 and 10Cu-22-mpg-C_3_N_4_-1 materials.

Powder X-ray diffraction (XRD) patterns of 22-mpg-C_3_N_4_-1 and Cu-22-mpg-C_3_N_4_-1 materials were obtained to further characterize the materials (Figure 6). The XRD patterns of 22-mpg-C_3_N_4_-1 showed two pronounced diffraction peaks centered at ca. 27.5° and 13.0°, corresponding to the (002) and (100) reflections that are due to the interlayer stacking of the graphite-like, conjugated aromatic systems and the in-plane structure repeating motif of trigonal N-linkages of tri-s-triazene in g-C_3_N_4_, respectively [5,30]. This result indirectly confirmed the formation of g-C_3_N_4_. While the XRD patterns of both Cu-22-mpg-C_3_N_4_-1 materials showed the prominent peak associated with C_3_N_4_, they did not show any diffraction peak associated with Cu species, due possibly to either the small size or the high dispersion of the Cu nanoparticles in them [28]. There is some evidence, at least, for the latter, based on TEM images (Figure 2e,f). Energy dispersive X-ray fluorescence (EDXRF) was carried out to further determine the amount of Cu in 10Cu-22-mpg-C_3_N_4_-1 material (Figure S2). The result showed that not only Cu was present in the material but also its amount was ca. 14.1 wt.%, which is close to what was used for its synthesis. Small angel X-ray scattering (SAXS) pattern of 22-mpg-C_3_N_4_-1 material (see Figure S3) showed a Bragg reflection in 2θ-region of 0.15°–0.75°, indicating the presence of some ordered pores with d-spacing of ca. 26.1 nm, which is also in line with the results obtained from TEM images and by N_2_ porosimetry.

**Figure 7:**
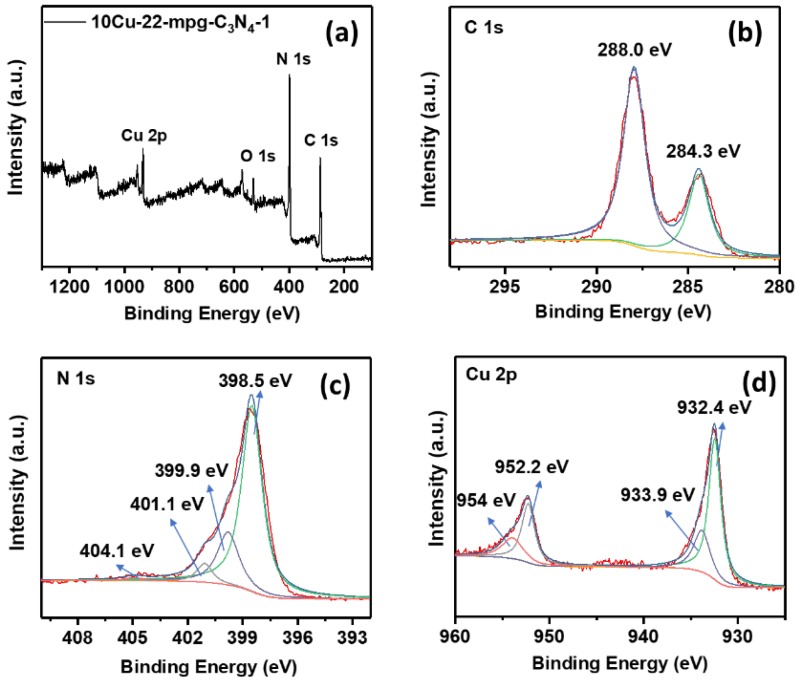
(**a**) XPS survey spectra of 10Cu-22-mpg-C_3_N_4_-1; High-resolution XPS spectra of (**b**) C 1s peak; (**c**) N 1s peak and (**d**) Cu 2p peak of 10Cu-22-mpg-C_3_N_4_-1.

X-ray photoelectron spectroscopy (XPS) analysis was performed to determine the composition of 10Cu-22-mpg-C_3_N_4_-1 material as well as the chemical states of the elements existing in it. The survey spectrum showed that only C, O, N and Cu were present in the material (Figure 7a). The C 1s peak in the spectrum showed two peaks centered at ca. 284.3 eV and 288.0 eV, corresponding to sp^2−^bonded carbon atoms of C–C and N–C=N groups, respectively (Figure 7b) [5]. The N 1s peak in the spectrum was deconvoluted into four peaks with binding energy values of 398.5, 399.9, 401.1 and 404.1 eV, which can be ascribed to sp^2^−bonded N atoms of C–N=C species, N atoms in tertiary N–(C)_3_ groups, N atoms in N–H moieties and π excitation, respectively (Figure 7c) [5,31]. The oxidation state of Cu was determined on the basis of the position of the Cu 2p_3/2_ peak (Figure 7d). Two peaks at binding energies of ca. 932.4 and 952.2 eV were observed, corresponding to Cu 2p_3/2_ and Cu 2p_1/2_ of Cu^0^ and/or Cu^1+^. The additional small peaks observed at ca. 933.9 and 954.0 eV could be assigned to Cu^2+^ species, which were likely formed from the oxidation of Cu^0^ on the surfaces of the Cu nanoparticles during exposure to air and humidity [32].

**Figure 8:**
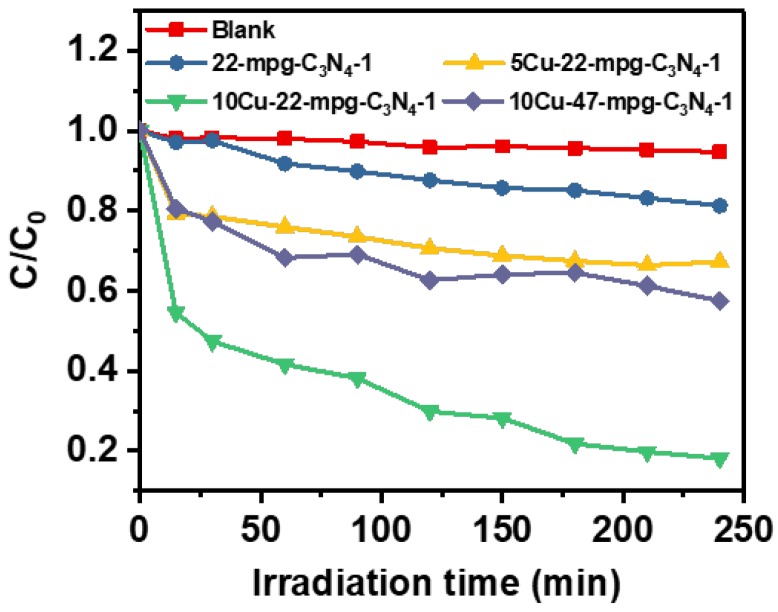
Degradation profiles of tartrazine yellow over the as-prepared mpg-C_3_N_4_ and Cu-mpg-C_3_N_4_ photocatalysts.

The photocatalytic activities of the materials for degradation of tartrazine yellow dye in solutions, a colorful additive that is widely used in various consumer products worldwide but is also often regarded as a cause of allergies, hyperactivity and even cancer, were evaluated (Figure 8). The necessary comparative and control experiments were also performed. In one control experiment, the extent of photolysis of tartrazine yellow without the presence of catalyst over an irradiation time of 240 min was found to be only ca. 5.3%. This revealed that the photodegradation of this dye in the absence of a catalyst was very slow. The photodegradation of the dye in the presence of a sample of 22-mpg-C_3_N_4_-1 was not good either, as only ca. 18.8% tartrazine yellow was photodegraded in the same time period. This suggested that as-synthesized mpg-C_3_N_4_ was not a good photocatalyst for the degradation of tartrazine yellow. However, the photocatalytic performance of the material for degradation of tartrazine yellow dye in solution was significantly improved after its surfaces were decorated with Cu nanoparticles, especially with about 10 wt.% of Cu nanoparticles. Specifically, the extents of photodegradation of tartrazine yellow dye in 240 min in the presence of 5Cu-22-mpg-C_3_N_4_-1 and 10Cu-22-mpg-C_3_N_4_-1 were found to be ca. 32.8% and ca. 81.9%, respectively, which were significantly higher than the one obtained in the presence of 22-mpg-C_3_N_4_-1. For comparison, the performance of 10Cu-47-mpg-C_3_N_4_-1 (a material that is synthesized using 47 nm silica nanoparticles as templates but containing the same amount, 10 wt.%, Cu nanoparticles) was also evaluated. This material resulted in ca. 42.5% photodegradation of tartrazine yellow in the same period of time. This material had a surface area is 87 m^2^/g, which is lower than that of 10Cu-22-mpg-C_3_N_4_-1 (i.e., 97 m^2^/g). These results suggest that, besides the surface-deposited Cu nanoparticles, higher surface area enhances the ability of Cu-doped mpg-C_3_N_4_ materials to photocatalytically degrade tartrazine yellow dye in solutions.

During photocatalytic degradation, efficient charge separation is important, because it allows the charges not to be compromised by undesirable charge recombination processes and to participate in various redox reactions. The available photogenerated electrons on the conduction band can then react with O_2_ and form superoxide radicals (O_2_^•−^). The resulting superoxide radicals as well as the photogenerated holes will help the photo-oxidative degradation of various compounds [2,33,34]. Unfortunately, plain g-C_3_N_4_ often suffers from rapid electron-hole recombination, and thus shows negligible photocatalytic activity. However, in the presence of metals such as Cu, which can trap the photogenerated electrons, the recombination rate of the photogenerated electron-hole pairs in g-C_3_N_4_ is significantly minimized. Hence, not surprisingly, the decoration of mpg-C_3_N_4_ with Cu nanoparticles facilitated the photodegradation of tartrazine yellow over the materials. In particular, 10Cu-22-mpg-C_3_N_4_ showed superior photocatalytic activity compared with plain 22-mpg-C_3_N_4_-1. Although 10Cu-47-mpg-C_3_N_4_-1 also showed better photocatalytic activity than plain 22-mpg-C_3_N_4_-1, its activity was inferior to that of 10Cu-22-mpg-C_3_N_4_-1 (the surface area of which is 97 m^2^/g); this was most likely due to its lower surface area (87 m^2^/g) and thus less exposed catalytic active sites [35]. Therefore, it can be said that, in addition to decorating its surfaces with Cu nanoparticles, introducing high surface area in mpg-C_3_N_4_ can improve the photocatalytic activity of g-C_3_N_4_ for degradation of tartrazine yellow dye. 

## 4. Conclusions

A facile synthetic route that produces highly photocatalytically active Cu nanoparticles-loaded mesoporous g-C_3_N_4_ (mpg-C_3_N_4_) from melamine using colloidal silica nanoparticles as hard templates has been developed. The synthetic conditions that produce mesoporosity in the materials have been investigated, and the mechanisms that lead to mesoporosity in the materials have been proposed. The surface area, pore size and pore volume of mpg-C_3_N_4_ could be tuned by changing the size of the silica nanoparticles used as templates to synthesize the materials. Modification of the surfaces of mpg-C_3_N_4_ by Cu nanoparticles has resulted in improved light absorption, charge separation, and photocatalytic activity over the materials. The resulting Cu nanoparticles-modified mpg-C_3_N_4_ (Cu-mpg-C_3_N_4_) materials were found to photocatalytically degrade tartrazine yellow dye, with activity much better than that of pure mpg-C_3_N_4_. In particular, the Cu-mpg-C_3_N_4_ material synthesized from melamine-to-silica nanoparticles mass ratio of 1:1 and then modified with 10 wt.% Cu showed superior photocatalytic activity toward the photodegradation of tartrazine yellow dye. The photocatalytic performances of the materials also depended on their specific surface area, besides the amount of Cu loaded on them. The present work provides a new synthetic strategy to prepare three-dimensionally interconnected porous g-C_3_N_4_ materials with large surface area, high porosity and great potential for remediation of common environmental pollutants.

## Figures and Tables

**Table 1:** Brunauer–Emmett–Teller (BET) surface area, average Barrett–Joyner–Halenda (BJH) pore size, and pore volume of different 12-mpg-C_3_N_4-*x*_ materials.

Size of SiO_2_ Nanoparticles (*y* = 12)	Mass Ratio of Melamine-to-SiO_2_ Nanoparticles (*x*)	BET Surface Area (m^2^/g)	Average Pore Size (nm) ^a^	Pore Volume (cm^3^/g)
12	- ^b^	3	- ^c^	0.04
12	0.25	7	- ^c^	0.06
12	0.5	110	6.2	0.22
12	1	136	11.7	0.27
12	4	9	- ^c^	0.05

^a^ Pore size is measured based on the adsorption data. ^b^ No colloidal silica nanoparticles were used in this case. Melamine was carbonized and the carbon materials obtained from it was characterized for comparison. ^c^ No definite value was obtained here, perhaps due to the largely non-porous structure in the material.

**Table 2:** BET surface area, average BJH pore size, and pore volume of different *y*-mpg-C_3_N_4_-1 materials.

Size of SiO_2_ Nanoparticles (*y*)	Mass ratio of Melamine-to-SiO_2_ Nanoparticles (*x* = 1)	BET Surface Area (m^2^/g)	Average Pore Size (nm) ^a^	Pore Volume (cm^3^/g)
12	1	136	11.7	0.27
22	1	118	22.5	0.56
47	1	92	43.6	0.88
79	1	73	ca. 80	1.05

^a^ Pore size is measured based on the adsorption data.

## Supplementary Materials

The following are available online at http://www.mdpi.com/2079-4991/8/9/636/s1.
