# Supplementary material for: Mesoporous Graphitic Carbon Nitrides Decorated with Cu Nanoparticles: Efficient Photocatalysts for Degradation of Tartrazine Yellow Dye
Source: Nanomaterials (Basel). 2018 Aug 21;8(9):636. doi: 10.3390/nano8090636 (PMC6163193; doi:10.3390/nano8090636)
Supplement: Supplementary file 1 [file nanomaterials-08-00636-s001.zip › nanomaterials-336919-SI.pdf]

Mesoporous graphitic carbon nitrides decorated with Cu nanoparticles: Efficient photocatalysts for degradation of tartrazine yellow

Tao Zhang[1], Isis P. A. F. Souza[2], Jiahe Xu[1], Vitor C. Almeida[2,*] and Tewodros Asefa[1,3,*]

[1] Department of Chemical and Biochemical Engineering, Rutgers, The State University of New Jersey, 98 Brett Road, Piscataway, New Jersey 08854, USA

[2] Laboratory of Environmental and Agrochemistry, Department of Chemistry, State University of Maringá, 5790 Colombo Avenue, Maringá, 87020-900, Paraná, Brazil

[3] Department of Chemistry and Chemical Biology, Rutgers, The State University of New Jersey, 610 Taylor Road, Piscataway, New Jersey 08854, USA

[*] Correspondences: (T.A.) E-mail: tasefa@chem.rutgers.edu; Tel.: +1-848-445-2970; (V.C.A.) E-mail: vcalmeida@uem.br; Tel.: +55 44 3011-4500

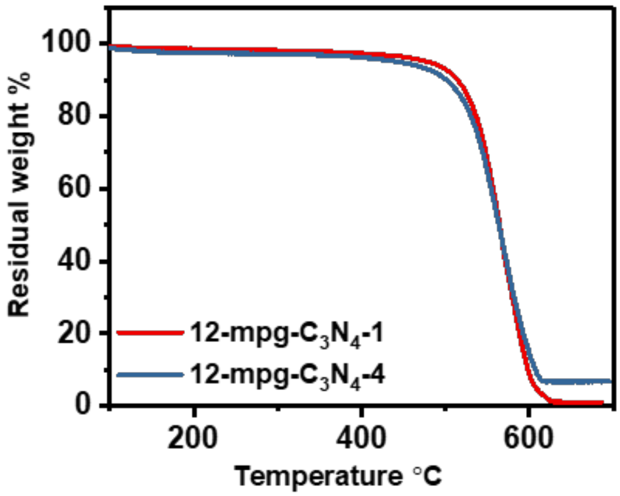

Figure S1. TGA curves obtained under air for 12-mpg-C_3N_4-1 and 12-mpg-C_3N_4-4 materials.

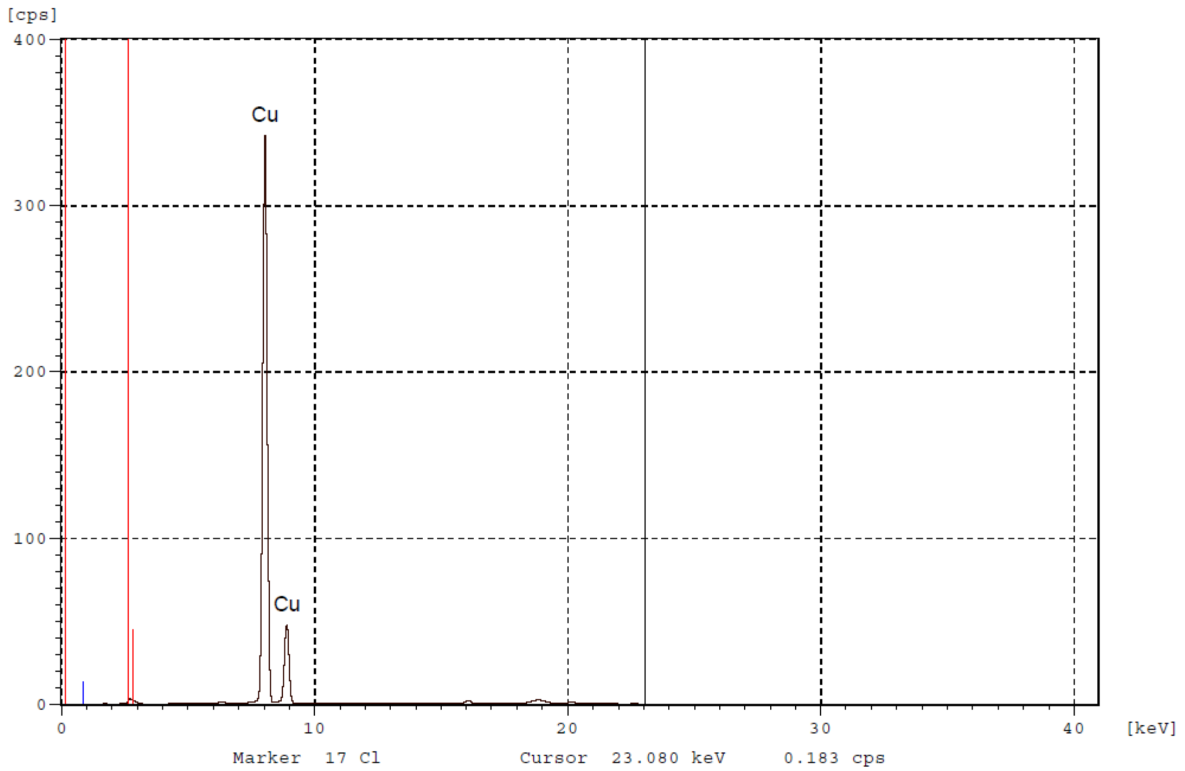

Figure S2. EDXRF results of 10Cu-22-mpg-C_3N4-1.

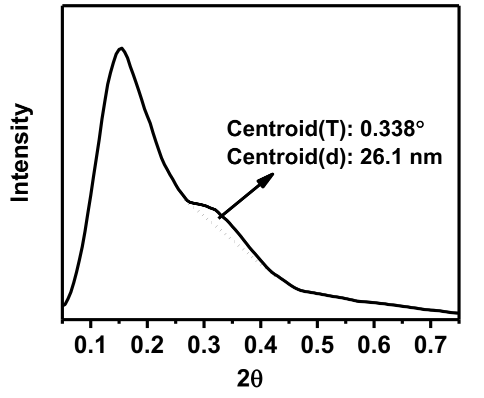

Figure S3. SAXS patterns of 22-mpg-C_3N_4-1.
